# Epitaxial Growth of Uniform Single-Layer and Bilayer Graphene with Assistance of Nitrogen Plasma

**DOI:** 10.3390/nano11123217

**Published:** 2021-11-26

**Authors:** Shaoen Jin, Junyu Zong, Wang Chen, Qichao Tian, Xiaodong Qiu, Gan Liu, Hang Zheng, Xiaoxiang Xi, Libo Gao, Can Wang, Yi Zhang

**Affiliations:** 1 National Laboratory of Solid State Microstructure, School of Physics, Nanjing University, Nanjing 210093, China; jsecumt@163.com (S.J.); nicole2020215@126.com (J.Z.); 15116118361@163.com (W.C.); tianqichao1995@outlook.com (Q.T.); mg1922065@smail.nju.edu.cn (X.Q.); lg@smail.nju.edu.cn (G.L.); MG1722068@smail.nju.edu.cn (H.Z.); xxi@nju.edu.cn (X.X.); lbgao@nju.edu.cn (L.G.); 2 Collaborative Innovation Center of Advanced Microstructures, Nanjing University, Nanjing 210093, China

**Keywords:** graphene, epitaxial, ARPES, band structure, Raman spectroscopy, nitrogen plasma

## Abstract

Graphene was reported as the first-discovered two-dimensional material, and the thermal decomposition of SiC is a feasible route to prepare graphene films. However, it is difficult to obtain a uniform single-layer graphene avoiding the coexistence of multilayer graphene islands or bare substrate holes, which give rise to the degradation of device performance and becomes an obstacle for the further applications. Here, with the assistance of nitrogen plasma, we successfully obtained high-quality single-layer and bilayer graphene with large-scale and uniform surface via annealing 4H-SiC(0001) wafers. The highly flat surface and ordered terraces of the samples were characterized using in situ scanning tunneling microscopy. The Dirac bands in single-layer and bilayer graphene were measured using angle-resolved photoemission spectroscopy. X-ray photoelectron spectroscopy combined with Raman spectroscopy were used to determine the composition of the samples and to ensure no intercalation or chemical reaction of nitrogen with graphene. Our work has provided an efficient way to obtain the uniform single-layer and bilayer graphene films grown on a semiconductive substrate, which would be an ideal platform for fabricating two-dimensional devices based on graphene.

## 1. Introduction

Graphene, an atomically single layer of carbon $sp^{2}$ bonded in a honeycomb lattice, has attracted enormous attentions of researchers due to its novel physics and broad application prospects [1,2,3]. Graphene can be used as an ideal substrate for the epitaxial growth of novel two-dimensional (2D) materials due to its native weak van der Waals interaction at the interface [4,5,6]. In addition, graphene shows great application potential in advanced devices. For examples, the single-layer graphene (SLG) photodetectors have the broadest photo response with high photoconductive gain 8.61 AW${}^{-1}$ [7]. The plasma-fluorinated SLG can be applied as a gas-sensing materials with extremely high sensitivity [8]. Besides, the SLG nanomechanical resonators have the advantage of more reproducible electrical properties and a larger surface area to capture incoming mass flux [9]. Based on the above facts, uniformly ordered growth of pure SLG on insulating substrate becomes necessary for preparing advanced nanodevices.

Many researches have demonstrated that the thermal decomposition of SiC(0001) is a feasible route to prepare manufacturable graphene films [1,10,11,12,13,14]. For example, the twisted bilayer graphene rotated 30${}^{{}^{\circ}}$ with its dodecagonal quasi-crystalline nature was realized on SiC(0001) surface [15]. Recently, Bocquet et al. have epitaxially grown a SLG in an unconventional orientation (*R*0${}^{{}^{\circ}}$) with respect to the SiC(0001) substrate in surfactant-mediated method [16]. As a platform, the epitaxial graphene on SiC(0001) can also be used to prepare 2D half-van der Waals metals by metal atomic intercalation [17,18]. Importantly, the different layers and stacking sequences of graphene can be selectively fabricated on SiC(0001) by controlling temperature [19,20,21]. Even though the graphene grown on Si-face SiC(0001) shows relatively better quality than that on C-face SiC(0001) [22], it is still difficult to precisely control the thickness of the grown graphene sample during the growth process. Especially, the growth of uniform SLG on SiC(0001) is still a challenge. In previous literatures [13,14], large-area synthesis of graphene was obtained by inert gas-assisted (N${}_{2}$, Ar) pressurization on SiC substrate. However, there are still coexisting mixtures of multilayer graphene islands and bare SiC substrate holes [13,14]. The mixture of different layers leads to the formation of domain boundaries, at which the particularly strong carrier scattering will result in high electronic resistance and degradation of device performance [23]. Importantly, the inhomogeneity of epitaxial graphene on SiC(0001) can affect photoemission spectropic experiments and result in controversial results for measuring band gap in previous literature [24,25,26,27].

Here, we provide an efficient way for selectively growing high-quality uniform SLG and BLG with the assistance of nitrogen plasma. Combing the scanning tunneling microscopy (STM), reflection high-energy electron diffraction (RHEED), angle-resolved photoemission spectroscopy (ARPES), and X-ray photoemission spectroscopy (XPS), we studied the surface morphology, band structure, and chemical bonding formation of these samples. Remarkably, the SLG and BLG grown under nitrogen plasma show a large-scale uniform surface morphology with regularly ordered terraces. Moreover, the Dirac bands of graphene treated with nitrogen plasma show sharper signals in the ARPRS spectra. These improvements were quantified from the analysis of momentum distribution curves (MDCs) in the ARPES spectra. Last, the results of XPS and ARPES spectra indicate that there is no intercalation or chemical reaction of nitrogen in SLG and BLG after treating in nitrogen plasma, showing the intrinsic characteristics of epitaxial graphene.

## 2. Materials and Methods

The samples were grown in a combined plasma-assisted MBE-STM ultra-high vacuum (UHV) system with a base pressure of 3.0 × 10${}^{-10}$ mbar. The Si-face SiC(0001) was chosen as the substrate as it is easier to grow high-quality graphene than the C-face SiC(000$\bar{1}$) [22]. The heating temperature was measured by a Photrix pyrometer with a measuring range between 149 ${}^{{}^{\circ}}$C and 2400 ${}^{{}^{\circ}}$C. The samples were transferred into the main chamber for ex situ ARPES and XPS measurements. The ARPES and XPS data were collected via a DA30L analyzer (SCIENTA OMICRON Inc., Danmarksgatan, Uppsala, Sweden). The ultraviolet (UV) light source was generated by a Helium lamp (FERMI Inc., Shanghai, China) with a monochromator for He I 21.2 eV (SPECS Inc., Berlin, Germany), and the monochromatic X-ray (SIGMA Inc., Cranberry Twp, PA, USA) was generated from an Al electrode excitation source (Al${}_{\alpha }$, 1486.7 eV). The light spot diameter is approximately 0.5 mm. During the ARPES and XPS measurements, the temperature of the samples were cooled down to 7 K by using a close-cycle cryogenerator. The energy resolution for ARPES and XPS measurements were better than 30 meV and 0.4 eV, respectively. The width and height of terrace on samples were determined by ex situ AFM measurements. The AFM measurements were performed with a Dimension Fastscan system (BRUKER Inc., Billerica, MA, USA) at tapping mode. The Raman spectroscopy measurements were all carried out at room temperature and the excitation source was a 532 nm laser (2.33 eV) with power of 0.5 mW which included a cryostation (MONTANA INSTRUMENTS Inc., Bozeman, MT, USA), and a grating spectro (TELEDYNE PRINCETON INSTRUMENTS Inc. Trenton, NJ, USA). More detailed experiments can be seen in Supporting Information.

**Figure 1:**
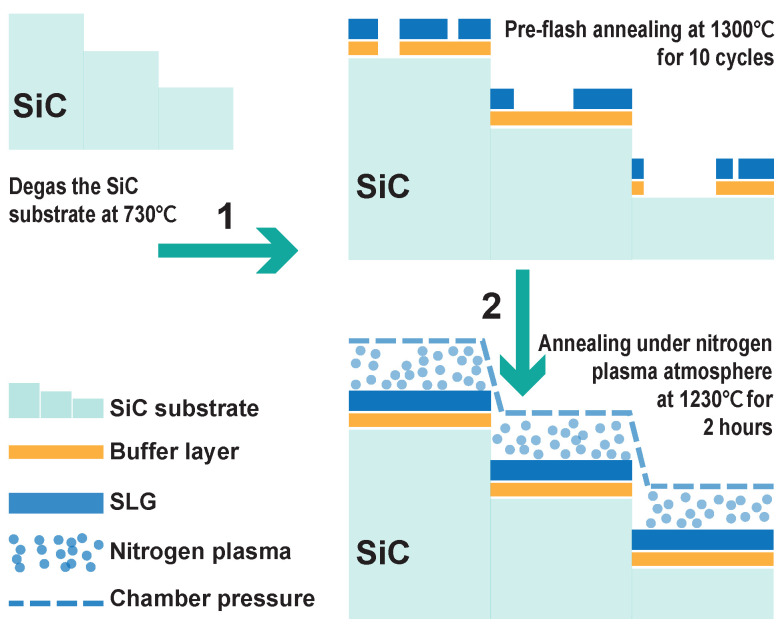
Schematic diagram of preparing uniform SLG on 4H-SiC(0001). The figure involves three main processes: the degassing of the substrate, pre-flash annealing, and annealing under nitrogen plasma atmosphere. The green arrows indicate the sequence of the three processes, and the lower left corner is the legend of the different lines and graphics. Among them, chamber pressure is to point out that the annealing pressure in nitrogen plasma atmosphere (1.1 × 10${}^{-5}$ mbar) is much higher than that of pre-flash annealing.

**Figure 2:**
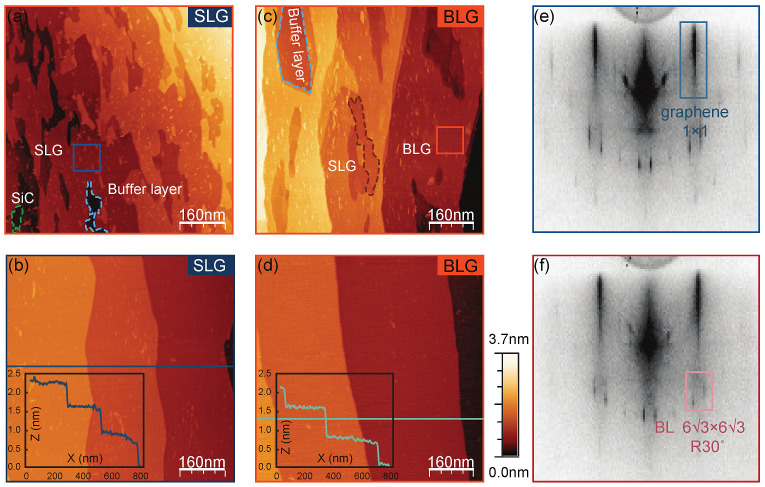
The changes of sample surface morphology through nitrogen plasma treatment. (**a**,**c**) STM images of SLG and BLG prepared by only flash annealing. The SiC and buffer layer coexisting with SLG are indicated by green and blue dashed line circles respectively in panel (**a**), the buffer layer and SLG coexisting with BLG are indicated by blue and brown dashed line circles respectively in panel (**c**). (**b**,**d**) STM images of SLG and BLG prepared by nitrogen plasma annealing after pre-flash annealing. The SLG and BLG show the consistent terrace height profiles (lower-left-corner insets in panels (**b**,**d**), respectively). (**e**,**f**) The RHEED patterns of SLG and BLG treated with nitrogen plasma, with electron beam along 〈11$\bar{2}$0〉 azimuth. Scanning parameters for STM: $V_{s}$ = 1 V, $I_{t}$ = 100 pA, room temperature.

The growth procedure of uniform SLG with assistance of nitrogen plasma is illustrated in Figure 1. First, the 4H-SiC(0001) substrate was heated at 730 ${}^{{}^{\circ}}$C for 1 h in the UHV chamber for degassing. Then, the pre-flash annealing process was carried out: using the resistance of the SiC substrate itself, the substrate was heated from room temperature to 1300 ${}^{{}^{\circ}}$C within 20 s by applying current (for more detailed description of the heating methods, see Figure S1 in Supporting Information). The sample maintained at 1300 ${}^{{}^{\circ}}$C for 30 s, then reduced to room temperature in 20 s. This pre-flash annealing cycle was repeated 10 times. Even though the vacuum in the first cycle might be slightly worse, the vacuum of the UHV chamber was finally maintained better than 1.0 × 10${}^{-9}$ mbar throughout the end of this process. Through pre-flash annealing procedure, we obtained a graphene sample with mixture of bare SiC surface, ($6\sqrt{3}\;\times \;6\sqrt{3}$) R30${}^{{}^{\circ}}$ reconstructed buffer layer and SLG [28,29,30,31], as can be seen from STM image in Figure 2a. The reconstructed buffer layer has a graphene-like honeycomb structure but shows $6\sqrt{3}\;\times \;6\sqrt{3}$ reconstruction of the graphene lattice constant and has been well studied with a undistoted σ-state but a distorted π-state [32].

The next step is critical for obtaining uniform SLG on 4H-SiC(0001). If we simply continue to repeat the pre-flash annealing cycles, BLG islands will appear on the 4H-SiC(0001) surface, forming a mixture of SLG and BLG islands. Therefore, in order to obtain a perfect uniform and pure SLG, the sample was then annealed under nitrogen plasma atmosphere. The nitrogen plasma was produced from a SPECS plasma source with power of 200 W and nitrogen pressure of 1.1 × 10${}^{-5}$ mbar. The pre-prepared substrate with few SLG domains was kept at 1230 ${}^{{}^{\circ}}$C for 2 h during exposing nitrogen plasma (the heating method was the same as the pre-flash stage and the heating rate was 10 ${}^{{}^{\circ}}$C/s). The preparation process of BLG samples is similar to that of SLG. The pre-flash annealing process for obtaining BLG was performed at 1350 ${}^{{}^{\circ}}$C and repeated for 60 cycles, resulting a mixture of SLG holes and BLG on the substrate surface. The annealing temperature of substrate under nitrogen plasma was 1300 ${}^{{}^{\circ}}$C for preparing BLG. The initial degassing process is as same as that of the preparation of SLG (see Figure 1).

## 3. Results and Discussion

In Figure 2, we compared the surface morphology between the samples with and without post-annealing process under nitrogen plasma using STM. The typical surface morphology of SLG and BLG produced by repeated flash annealing process are shown in Figure 2a,b, respectively. Obviously, it is difficult to precisely control the thickness of the grown graphene using flash annealing process method. In addition to SLG, the buffer layer and bare SiC substrate holes can also be seen in Figure 2a. The $\left(6\sqrt{3}\;\times \;6\sqrt{3}\right)$ R$30^{{}^{\circ}}$ reconstructed buffer layer forms the intrinsic interface structure between epitaxial graphene and SiC substrate. Meanwhile, the substrate terraces can be roughly identified. Different from the clean step edges of the raw SiC substrate (see Figure S2 in Supporting Information), we observed that the adjacency of these terraces is ambiguous and shows complex finger shapes as the reports in previous literature [11,33,34]. The morphology of the BLG prepared by pre-flash annealing shows relative distinct boundaries of the terraces (see Figure 2b). However, the BLG areas still coexist with the uncovered regions of SLG. The additional height information of the buffer layer, SLG, and BLG are shown in Figure S3, Supporting Information.

As a comparison, the surface morphologies of SLG and BLG prepared by annealing under nitrogen plasma atmosphere after pre-flash annealing are shown in Figure 2c,d, respectively (see Figure S4 and Figure S5 in Supporting Information for the atomic resolution and more large-size STM images). Apparently, the nitrogen plasma treated SLG and BLG samples both show a uniform morphology, demonstrating a high degree of consistency with the height and width of the highly ordered terraces. The lines profile in Figure 2c,d indicates that each terrace height is ~0.75 nm, which corresponds to the height of three SiC double layers [10,35]. The average width of each terrace is approximately 300∼400 nm, which is approximately three times wider than that of the raw SiC(0001). These indicate that during the growth process, three SiC terraces merged into one, similar to previous report [10]. Moreover, the sharp patterns of RHEED in Figure 2e,f show the high quality of the samples. Notably, there is no significant difference between the RHEED patterns of SLG and BLG, except for the ($6\sqrt{3}\;\times \;6\sqrt{3}$) R30${}^{{}^{\circ}}$ reconstruction of SLG is relatively more clearly showed in RHEED patterns compare to that of BLG. The detailed comparison of the RHEED patterns can be seen in Figure S6, Supporting Information.

**Figure 3:**
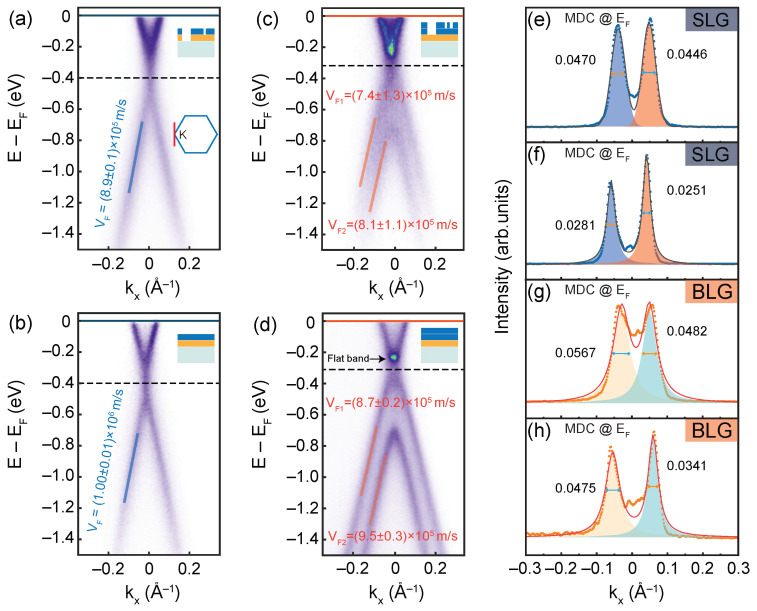
Band dispersion spectra of π bands around the K point of the Brillouin zone (see red line in the inset) via the ARPES spectra of epitaxial graphene on 4H-SiC(0001) and the linear fitting results of (**a**) SLG without nitrogen plasma treatment, (**b**) SLG with nitrogen plasma treatment, (**c**) BLG without nitrogen plasma treatment, and (**d**) BLG with nitrogen plasma treatment, respectively. The positions of Dirac points in panel (**a**–**d**) are marked with black dashed lines. The flat band is pointed out with a black arrow in panel (**d**). The Fermi velocities with the error of 95% confidence bounds are also marked in the panels (**a**–**d**). (**e**–**h**) MDCs measured along the Fermi level corresponding to the panels (**a**–**d**), the FWHM is marked on each panel.

We further explored the effect of nitrogen plasma on the band dispersion of SLG and BLG by using ARPES in Figure 3. In our ARPES spectra, the Dirac points of nitrogen plasma-treated SLG and BLG samples display a typical behavior of epitaxial graphene on SiC substrate, see Figure 3b,d. For the SLG sample, the Dirac point is ~400 meV below the Fermi level, while that for the BLG sample is ~300 meV below the Fermi level. The energy position of Dirac points is identical to the previous reported measured by ARPES [20,24,36,37,38], which is due to the charge transfer between substrate and graphene. Meanwhile, the position of Dirac points in nitrogen plasma-treated SLG and BLG coincides with that of SLG and BLG directly prepared without nitrogen plasma in UHV, as can be seen in Figure 3a,c. After N plasma treatments, the position of Dirac points does not move compared to the untreated ones, which indicates that N plasma treatments can not introduce N element doping [39,40,41]. In addition, the ARPES spectra in Figure 3b,d clearly indicates the AB stacking type of the BLG [42].

We performed linear fit to the experimental data from energy distribution curves (EDCs) peak position, as shown in Figure 3a–d. The corresponding stacked EDCs are given in Figure S7, Supporting Information. The Fermi velocity of the Dirac electron in graphene is proportional to the slope of the Dirac cone. Therefore, the Fermi velocity can be obtained by using $\nu_{F}\;=\;\left(1/\hbar \right)\left(\partial E/\partial k\right)$, where *ℏ* is the reduced Planck constant. The Fermi velocity of SLG untreated with nitrogen plasma is $\nu_{F}\;=\;\left(0.89\;\pm \;0.01\right)\;\times \;10^{6}$ m/s in Figure 3a, while it is $\nu_{F}\;=\;\left(1.00\;\pm \;0.01\right)\;\times \;10^{6}$ m/s for SLG-treated nitrogen plasma in Figure 3b, the errors is in 95% confidence bounds that from the linear fitting function of MATLAB. It can be concluded that the Fermi velocity of the Dirac electrons in SLG and BLG processed with nitrogen plasma annealing becomes larger than that of the untreated samples. In detail, for SLG, the Fermi velocity of Dirac electrons after nitrogen plasma treatment is ~12% higher than that before treatment. For BLG, the Fermi velocities of the upper and lower Dirac cones increase by 18% and 17%, respectively (see Figure 3c,d). In addition, the slope of the lower Dirac cone is larger than the slope of the upper Dirac cone in BLG; this phenomenon occurs regardless of whether the sample is treated with nitrogen plasma or not. For the untreated sample, the slope of the lower Dirac cone is 10% higher than that of the upper. While the sample treated with nitrogen plasma, the slope of the lower Dirac cone is 9% higher than that of the upper. All the values of Fermi velocities are in the same order of magnitudes to the previous reports [43,44].

The Dirac bands of ARPES spectra in Figure 3b,d show a sharper and narrower dispersion than that of Figure 3a,e. Especially, we observed a clear flat band in BLG treated with nitrogen plasma. This sharp flat band is the result of interlayer coupling and sublattice effect in BLG on SiC(0001) [45]. Figure 3e–h further confirms the band dispersion modification in the cuts of momentum distribution curves (MDCs) at the Fermi level. The energy band difference before and after treatment by nitrogen plasma can be quantified from the full width at half maximum (FWHM) of the peaks by fitting the MDCs to Lorentzian peaks. We analyzed and compared the results of FWHM in Figure 3e–h. The values of FWHM can be seen from Figure S8, Supporting Information. The average FWHM of SLG untreated with nitrogen plasma is ~0.045 (Å${}^{-1}$) (Figure 3e), while narrows to ~0.026 (Å${}^{-1}$) (Figure 3f) after being treated by nitrogen plasma. For BLG samples, the average FWHM is ~0.052 (Å${}^{-1}$) for that untreated with nitrogen plasma (Figure 3g), while it narrows to 0.041 (Å${}^{-1}$) (Figure 3h) after being treated with nitrogen plasma. The FWHM of the left side peak in SLG treated with nitrogen plasma is only ~60% of that of untreated sample, while the right one is only ~6%. In other words, the band of SLG treated with nitrogen plasma becomes sharper, specifically, the average width is only ~58% of that of untreated SLG. Correspondingly, the BLG treated with nitrogen plasma has an average energy band width of 77% compared with that of the untreated BLG (the FWHM on the left is 84%, the right one is 71%). The FWHM of the band signals in the ARPES measurements are mainly dictated by the imaginary part of the self-energy in the spectral function, which represents the single-particle scattering rate and could be an identifier of the sample surface quality [46]. The FWHM broadening in Figure 3e,h may result from the scattering from the terrace edges, boundaries, and islands of the rough surface, as can be seen in Figure 2a,b. The sharper band and narrower FWHM in ARPES spectra indicate the improvement of sample quality after being treated with nitrogen plasma.

**Figure 4:**
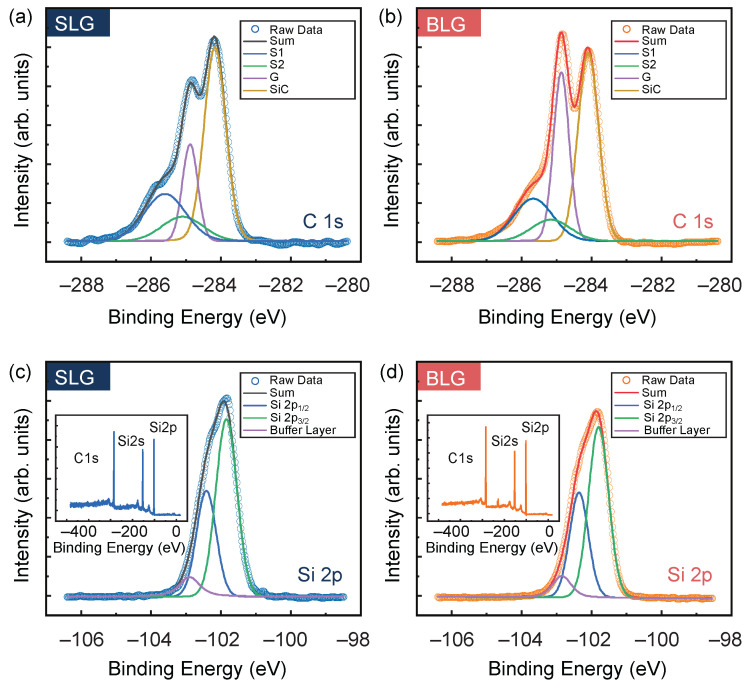
Chemical bonding spectra measured via XPS. (**a**,**b**) High resolved C 1s core-level spectrum of SLG and BLG treated with nitrogen plasma, the spectrums contain the deconvolution of SiC substrate (marked as SiC), the ($6\sqrt{3}\;\times \;6\sqrt{3}$) R$30^{{}^{\circ}}$ buffer layer, and the graphene layers (marked as G). (**c**,**d**) High resolved Si 2p core-level spectrum of SLG and BLG treated with nitrogen plasma, the spectrums contain contributions from spin–orbit split doublets, and the bonding between buffer layer and Si-terminated surface of SiC. Insets in panels (**c**,**d**) are the wide range XPS spectra of SLG and BLG treated with nitrogen plasma, respectively.

In order to examine the interaction between graphene and nitrogen plasma, we conduct XPS measurements on our samples in Figure 4. The C 1s core-level spectra of SLG and BLG treated with nitrogen plasma show a characteristic feature of the epitaxial graphene growth on SiC (see Figure 4a,b). The peaks S1 (285.6 eV) and S2 (285.1 eV) in C 1s core-level spectra are the buffer layer related components. The peak labeled by SiC at 283.9 eV, corresponds to the carbon atoms of the 4H-SiC(0001) substrate. The pristine $sp^{2}$-hybridized carbon of epitaxial graphene (labeled G) located at 284.7 eV. The energy position of C 1s core-level in agreement with samples prepared in previous literature [13,47,48,49,50]. The G peaks of SLG and BLG differ only in intensity, and the area ratio of the G peak and SiC peak is ~0.34 for SLG film, while the ratio is ~0.70 for BLG film, which is approximately twice. There is no difference between the Si 2p core-level spectra of SLG and BLG, both of them are made up of spin-orbit split doublets, which are consistent with the in UHV-grown films [47,50]. Note that no additional components are observed in either C 1s or Si 2p XPS spectra. This indicates that nitrogen does not bond with graphene or Si in SiC substrate. There is no signal of N in the wide range XPS spectra (see the insets of Figure 4c,d), and the high-resolved spectra around the energy of N 1s core level (see Figure S9, Supporting Information).

**Figure 5:**
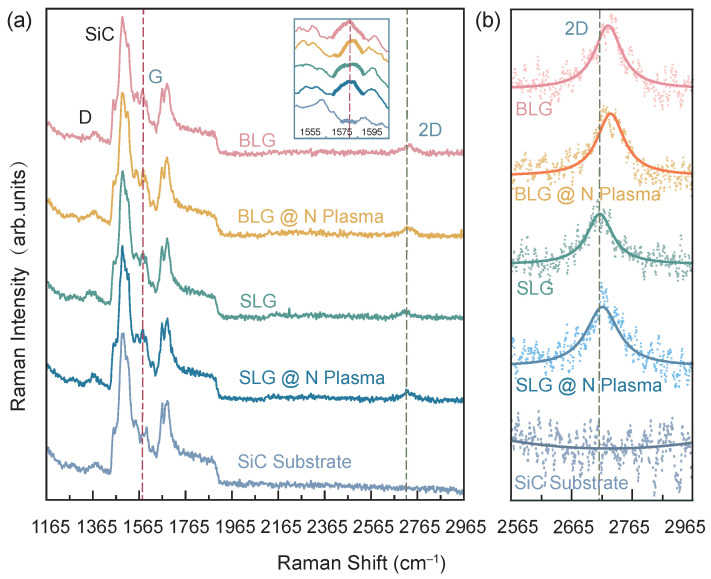
Raman spectra of epitaxial graphene on SiC and SiC substrate. (**a**) Raman spectra of BLG without and with N plasma treatments (denoted as “BLG@N plasma” in the figure), SLG and SLG without and with N plasma (denoted as “SLG@N plasma” in the figure), and Si-face 4H-SiC(0001) substrate. The inset is an enlarge part of the G band region of spectra in panel (**a**). (**b**) High-resolved 2D band of BLG without and with N plasma, SLG without and with N plasma treatments, and Si-face 4H-SiC(0001) substrate, respectively. The dot symbols are experimental data and the solid lines are the fitted curves. There is a shift of ~20 cm${}^{-1}$ between the 2D band of SLG and BLG.

In Figure 5, we show the Raman spectra at room temperature of epitaxial graphene on SiC(0001) measured by laser with 532 nm. There is a shift of ~20 cm${}^{-1}$ between the 2D bands of SLG and BLG, which has also been mentioned in the previous report [51]. In addition to the shift of the 2D bands, the previous report also mentioned that the 2D band of BLG is broader than the 2D band of SLG, which is not obvious in our Raman spectra measurements. Note that the defect-induced D band of BLG has a sharp increase in intensity compared to that of SLG and SiC due to intervalley scattering in the previous report [51]. These results indicate that their BLG samples are poorly crystalline, which may have caused the 2D band of BLG to be boarder. In our graphene samples, the D band intensities are consistently very weak, which might be the reason that we only observed the 2D band shifted but not broadened. In fact, the D peak of the graphene samples always maintain the same intensity as the SiC substrate, considering that Raman spectroscopy is not a surface-sensitive method, but it usually measures deeper inside to a larger volume [52]; the D peak in the graphene samples is more likely to come from the substrate. In addition, the N doping would provoke a blue shift of the 2D Raman band due to the charge transfer between the SiC substrate and the graphene [53,54], which was not observed in our Raman measurements. Therefore, both the Raman and XPS measurements results also indicate that there is no nitrogen doping, which are consistent with the conclusion obtained in our previous ARPES results.

Why is the surface morphology of the sample prepared by flash annealing not highly improved after being annealed in nitrogen plasma atmosphere? Under the UHV condition, the diffusion of Si is not limited, and the rate cannot be well controlled, resulting in the uncontrollable rate of graphene films generation. Similar to the effect of nitrogen and argon in reducing the sublimation rate of Si [13,14], the nitrogen plasma could limit the sublimation rate of Si with the relative low pressure. During the thermal decomposition process of SiC, Si atoms sublimate and desorb from the SiC substrate, then the released carbon atoms diffuse and rearrange to form buffer layer. When the Si atoms are continuously desorbed, a new ($6\sqrt{3}\;\times \;6\sqrt{3}$) R30${}^{{}^{\circ}}$ reconstruction interlayer is formed under the original buffer layer, and then the original buffer layer becomes SLG [55,56]. In the annealing process of SLG under the nitrogen plasma atmosphere, where the sample surface is covered with one layer of graphene, it reaches an equilibrium at the specific heating temperature and the chamber pressure. In this equilibrium, the Si in the SiC substrate cannot be sublimated, which means that new graphene multilayers cannot be generated. While the for the uncovered area, Si atoms can still be sublimated. The newly generated graphene fills these uncovered areas and finally forms a uniform SLG film. For the BLG, the establishment of this equilibrium requires a higher temperature of 1300 ${}^{{}^{\circ}}$C. Finally, we also noticed that although the SLG has not fully covered the substrate after flash annealing, small islands appear on the SLG (see Figure S3, Supporting Information). These islands disappear after being annealed under a nitrogen plasma atmosphere. This may be due to the convex surface islands are polished under the collision and friction of high-energy nitrogen atoms at high temperature [57,58,59].

## 4. Conclusions

In summary, we introduced a practical and efficient method for preparing uniform graphene by thermal decomposition of 4H-SiC(0001) with the assistance of nitrogen plasma. With the help of nitrogen plasma, we can precisely control the number of graphene layers. We prepared large-scale uniform and pure SLG and BLG on 4H-SiC(0001) substrate, which have perfect surface morphology with regular step height of approximately 0.75 nm. The ARPES and XPS measurements indicate the high quality of our samples, and there are no nitrogen atoms chemically bonded, intercalated, or doping with graphene. The key to the formation of a uniform surface lies in the equilibrium of the sublimation of Si, at the same time, the nitrogen plasma may have the effect of polishing the protruding islands on the sample surface. Our work provided an efficient way to improve the quality of the epitaxial graphene and offered a platform to epitaxial growth of 2D materials based on graphene.

## Data Availability

The data presented in this study are available on request from the corresponding author.

## Supplementary Materials

The following are available online at https://www.mdpi.com/article/10.3390/nano11123217/s1. Details of methods for ARPES, XPS, and Raman spectroscopy experimental techniques, heating method (Figure S1), AFM image of 4H-SiC(0001) substrate (Figure S2), STM images for SLG and BLG without nitrogen plasma treatment (Figure S3), atomic resolution STM for SLG and BLG (Figure S4), large-area STM images (Figure S5), the analysis of RHEED patterns (Figure S6), EDCs of ARPES spectra (Figure S7), FWHM of MDCs at the Fermi level (Figure S8), and XPS N 1s core-level (Figure S9).
