# Supplementary material for: Epitaxial Growth of Uniform Single-Layer and Bilayer Graphene with Assistance of Nitrogen Plasma
Source: Nanomaterials (Basel). 2021 Nov 26;11(12):3217. doi: 10.3390/nano11123217 (PMC8706778; doi:10.3390/nano11123217)
Supplement: Supplementary file 1 [file nanomaterials-11-03217-s001.zip › nanomaterials-1451490-supplementary.pdf]

Supplementary Materials for: Epitaxial Growth of Uniform Monolayer and Bilayer Graphene with Assistance of Nitrogen Plasma

Shaoen Jin [1], Junyu Zong [1], Wang Chen [1], Qichao Tian [1], Xiaodong Qiu [1], Gan Liu [1], Hang Zheng [1], Xiaoxiang Xi [1,2], Libo Gao [1,2], Can Wang [1,2]* andYi Zhang [1,2]*

1 National Laboratory of Solid State Microstructure, School of Physics, Nanjing University, Nanjing 210093, China
2 Collaborative Innovation Center of Advanced Microstructures, Nanjing University, Nanjing 210093, China
* Correspondence: jerrywang@nju.edu.cn (C.W.); zhangyi@nju.edu.cn (Y.Z.).

A. Samples heating method.

We use the resistance of the SiC itself by applying direct current for heating. The schematic diagram of the sample loading mode during heating is shown in Figure S1. We built up a close-loop temperature control system consisted of a programmable power supply, an infrared pyrometer, and a self-designed software based on PID algorithm to precisely control the sample heating temperature.

B. AFM of SiC substrate.

The AFM image of the raw 4H-SiC(0001) substrate is shown in Figure S2. The width of the SiC terraces approximately corresponds to the width of the graphene terrace after the N plasma treatments.

C. Morphology of pre-ashing samples.

The graphene films prepared by flash annealing have mixed layers, and different layers are identified with height information. It can be seen from the height profile inserted in Figure S3 that the heights of buffer layer and MLG are different, which are about 1.6 Å and 3.0 Å respectively, this is close to the previous research results.[1,2] In addition, some islands were found on the MLG films, they have two main heights, one is about 3.5 Å, the other is about 7 Å. The former may be a small piece of bilayer graphene, when considering the height of latter is close to 7.5 Å. It may be initially connected to the silicon carbide step (Bottom-up, three SiC double layers, buffer layer and MLG), but later isolated due to erosion mechanism during the formation of graphene.[3,4] The height of the BLG is about 5.5 Å, derived from 2.5 Å first graphene layer and 3 Å second graphene layer.[1]

D. Atomic resolution STM images of N plasma treated graphene.

The atomic resolution STM images of both monolayer and bilayer graphene with the N plasma treatments are shown in Figure S4, from which can be seen that N plasma treatments do not change the atomic arrangement of graphene, but only modify the surface morphology and reduce defect density.

E. Wide area and large size STM images.

In order to verify that the substrate is regularly covered in a large range, we performed a large-size STM scan at the center and four corners of a 3.3×3.3 μm^2 area as shown in Figure S5, and it can be seen that both MLG and BLG with N plasma treatments exhibit a uniform and flat morphology. Furthermore, by changing the position of the sample holder, we can change the relative position of the STM tip to the sample by a millimeter scale level, and then repeat the above scanning result, so that we were able to verify the complete coverage of a larger range. Therefore, it can be concluded that this is a phenomenon that occurs in a long-range order.

Citation: Jin, S.; Zong, J.; Chen, W.; Tian, Q.; Qiu, X.; Liu, G.; Zheng, H.; Xi, X.; Gao, L.; Wang, C.; Zhang, Y. Epitaxial Growth of Uniform Monolayer and Bilayer Graphene with Assistance of Nitrogen Plasma. *Nanomaterials* **2021**, *11*, 3217. https://doi.org/10.3390/nano11123217

Received: 21 October 2021
Accepted: 24 November 2021
Published: 26 November 2021

Publisher's Note: MDPI stays neutral with regard to jurisdictional claims in published maps and institutional affiliations.

F. RHEED analysis.

At first glance, the intensity of spots from buffer layer in bilayer graphene RHEED patterns is weaker. We qualitatively demonstrated the visual difference in the RHEED patterns of monolayer and bilayer graphene in Figure S6. In RHEED patterns of single-layer graphene, the ratio of the spot intensity from graphene and buffer layer to the spot intensity from only buffer layer is 2.34, while in the RHEED patterns of bilayer graphene the ratio is 2.08, a decrease of 11.11%. The RHEED description of graphene and buffer layer is in reference.[5]

G. The FWHM of MDCs.

Regardless of whether it is a MLG or BLG sample, the ARPES band images of the sample annealed under nitrogen plasma atmosphere are much sharper than that of the sample annealed without nitrogen plasma. We use the full width at half maximum (FHWM) of MDCs at the Fermi level to quantify this index. Figure S8 can more intuitively show that the improvement of our sample quality has significantly improved the quality of ARPES images. It can be seen from the figure that the blue and yellow triangle icons are much higher than the circular icons, and the values of the left and right peaks of the single layer and the double layer are very close. For the yellow icon, the value that the triangle filled on the left is higher than the circle is very close to the value that the triangle filled on the right is higher than the circle, and the same is true for the blue icon.

H. Raman spectroscopy measurements.

Raman spectroscopy measurements were all carried out at room temperature, the excitation source was a 532 nm laser (2.33 eV) with power of 0.5 mW to avoid laser induced surface heating. The Raman spectrum of epitaxial graphene is significantly different from that of exfoliated graphene on SiO_2/Si. For the exfoliated graphene, what monolayer graphene and bilayer graphene performed in the Raman measurements are very different. Specifically, the Raman spectrum of monolayer graphene shows a single and symmetrical 2D band, the relative band height ratio between 2D and G bands is about 4:1; while bilayer graphene shows a single a wide asymmetric 2D bands, the band height ratio between the 2D band and the G band is about 1:1.[6] However, for the epitaxial graphene on SiC, the intensity of the 2D band is relatively weak, and the difference between monolayer and bilayer graphene is relatively small.

I. Discussion on the absence of N doping.

In order to more accurately verify the absence of the N element in the as-grown samples after the N plasma treatments, we performed XPS measurements regarding N1s core level of both as-grown MLG and BLG after N plasma treatments, and no peaks were found even after multiple integrations as shown in Figure S9. Emilio Velez-Fort et al. have reported on the slight N doping of epitaxial graphene on 4H-SiC(0001), [7] their work shows that even a slight nitrogen doping will be reflected in the result of N 1s core level peak in XPS measurement. In addition, due to the charge transfer introduced by the N element, it will also cause the Dirac point to move 0.3 eV to a higher binding energy and will cause the (second order) 2D band to move to the right in the Raman measurements.[8] As we stated in the main text, whether monolayer or bilayer graphene, the band shift is not found after N plasma treatments. In addition, in our supplementary Raman measurements (see in Figure 5 in main text), no shift of the 2D band of the sample after N treatment was found. Therefore, the measurement results of ARPES, XPS and Raman mutually confirm that nitrogen is absent in the graphene. The graphene epitaxy process of Emilio Velez-Fort et al. is carried out in a nitrogen atmosphere. N atoms may replace C atoms during the formation of graphene, and may also form bonds with Si on the SiC surface,[9] thus forming low nitrogen doping. In order to avoid this situation, the epitaxial growth process of our graphene has been thoroughly completed in an ultra-high vacuum of 1.0×10^{-9} mbar before the N plasma treatments, eliminating the opportunity of N atoms doping. The N 1s core level XPS measurements and the π bands ARPES measurements also confirm our conclusion.

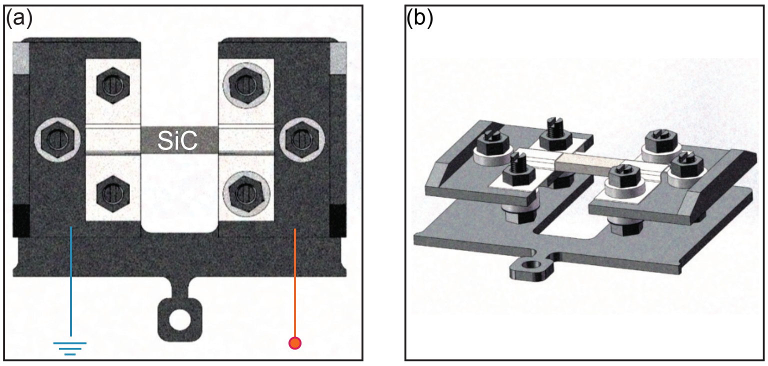

Figure S1. Schematic diagram of the SiC(0001) substrate loading and the heating method. (a) The SiC substrate is pressed on the molybdenum electrode plate by the tantalum sheet, the left electrode plate is grounded, and the right electrode plate is forward biased. When the power is turned on, the current flows from the right plate through the SiC substrate to the left plate, so as to achieve heating.(b) Side view of panel (a).

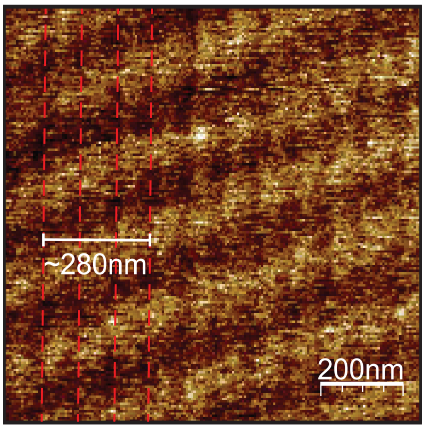

Figure S2. AFM image of raw SiC substrate. The width of the three SiC terraces is 280 nm, which corresponds to the width of one terrace of graphene treated with nitrogen plasma. The edge of the terrace is marked by red dashed line.

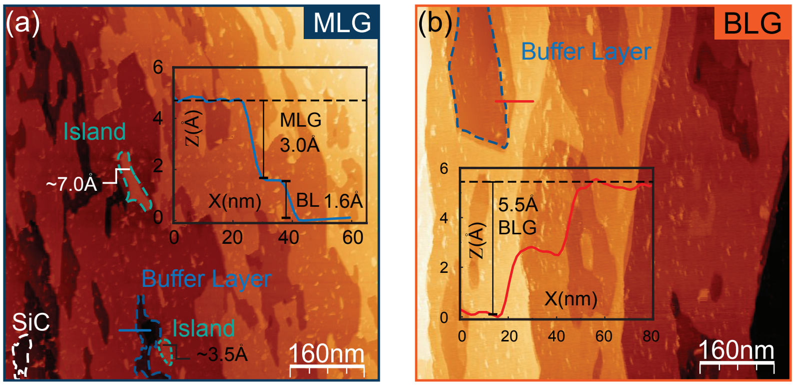

Figure S3. The surface morphology images of graphene films prepared by pre-flash annealing. (a), monolayer graphene surface morphology imaged by STM, the height profile of BL and MLG is shown in the inset of image (a), the blue line indicated the direction of measurement. The two of the islands are circled by green dashed line. (b), bilayer graphene surface morphology imaged by STM, the inset of image (b) shows a height profile along the red line in image (b).

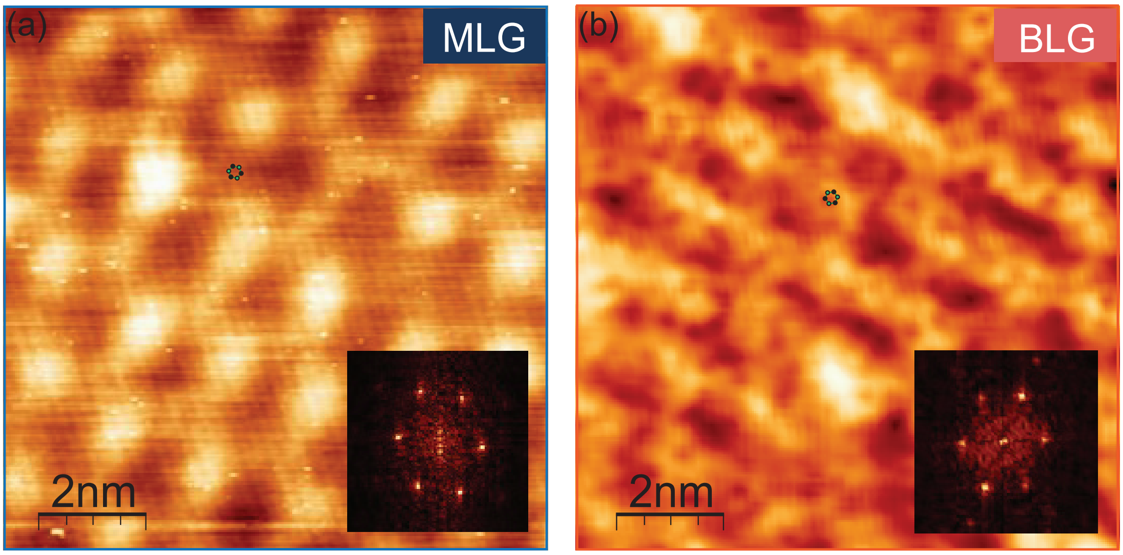

Figure S4. Atomic resolution STM images of (a) MLG and (b) BLG with N plasma treatments. The size of the images are both 10×10 nm^2, and the atom positions are marked in the images. Scanning parameters for STM: V_s = 1 V, I_t = 100 pA, room temperature.

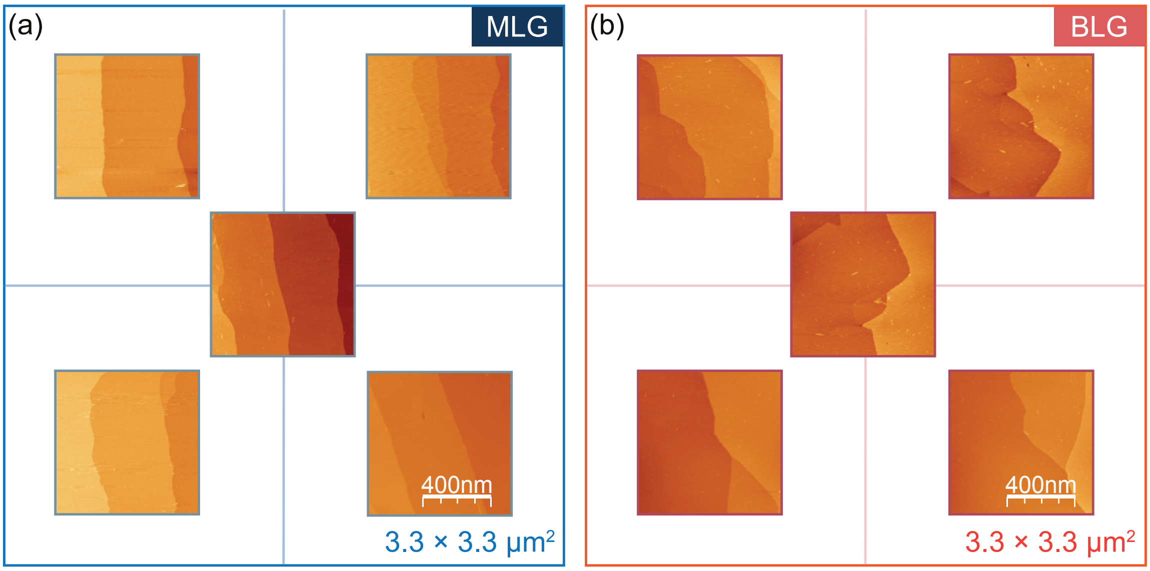

Figure S5. Wide area and large size STM scanned images of (a) MLG with N plasma treatments and (b) BLG with N plasma treatments. The STM collection range is a square area with a length of 3.3 μm, each sample was scanned a 0.8 μm STM image on the top, bottom, left, right and center of the square area. Scanning parameters for STM: V_s = 1 V, I_t = 100 pA, room temperature.

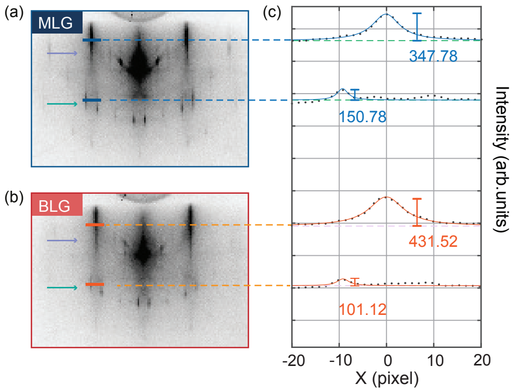

Figure S6. (a, b) RHEED patterns of MLG and BLG annealed in the nitrogen plasma. The purple arrows indicate the features from graphene and BL, and the green arrows indicate the features from BL. (c) Intensity distribution profiles taken along the lines in panel (a) and (b), the dotted lines represent the row data with fitting results (indicated by blue and orange solid lines) ride on. The dashed line (green and yellow) in panel (c) represents the backgrounds.

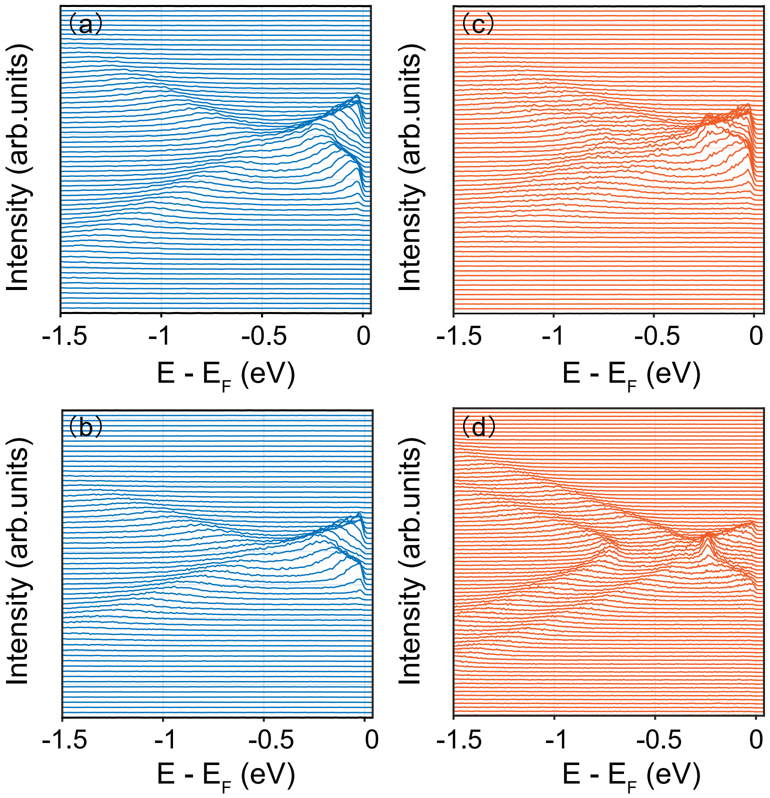

Figure S7. Energy distribution curves (EDCs) of ARPES spectra of (a) MLG without nitrogen plasma treatment, (b) MLG with nitrogen plasma treatment, (c) BLG without nitrogen plasma treatment, (d) BLG without nitrogen plasma treatment.

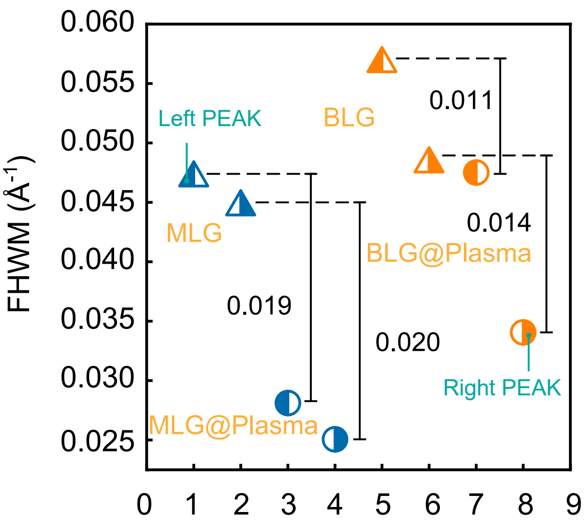

Figure S8. The scatter plot of FHWM shown in Figs. 3(e)-(h) in the main text. The blue graphic represents single-layer graphene, and the yellow represents double-layer graphene. The triangle represents the graphene prepared by pre-flash annealing, and the circle represents the graphene annealed in the nitrogen plasma atmosphere after pre-flash annealing. The color filling on the left and right of the graph corresponds to the peaks on the left and right, respectively.

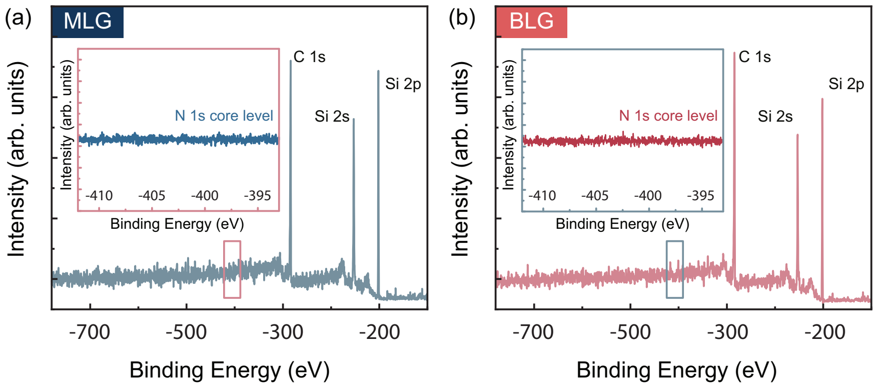

Figure S9. Verify the absence of nitrogen element by high resolved XPS measurements. Wide range XPS spectra of (a) MLG and (b) BLG after treatments of N plasma. High resolved N 1s core-level spectra of MLG [inset of panel (a) and BLG [inset of panel (b)] treated with N plasma demonstrate that there is no N doping in the samples. The N 1s core-level spectra of MLG and BLG after N plasma treatments are the integration results of 20 measurements in the same binding energy range (as shown by the boxes on the wide range XPS spectra, around -412 to -393 eV).

References

1. Rutter, G.M.; Crain, J.N.; Guisinger, N.P.; Li, T.; First, P.N.; Stroscio, J.A. Scattering and Interference in Epitaxial Graphene. *Science* **2007**, *317*, 219–222. doi:10.1126/science.1142882.
2. Razado-Colambo, I.; Avila, J.; Vignaud, D.; Godey, S.; Wallart, X.; Woodruff, D.P.; Asensio, M.C. Structural determination of bilayer graphene on SiC(0001) using synchrotron radiation photoelectron diffraction. *Sci. Rep.* **2018**, *8*. doi:10.1038/s41598-018-28402-0.
3. Bolen, M.L.; Harrison, S.E.; Biedermann, L.B.; Capano, M.A. Graphene formation mechanisms on $4H$-SiC(0001). *Phys. Rev. B* **2009**, *80*, 115433. doi:10.1103/PhysRevB.80.115433.
4. Wang, Q.; Zhang, W.; Wang, L.; He, K.; Ma, X.; Xue, Q. Large-scale uniform bilayer graphene prepared by vacuum graphitization of 6H-SiC(0001) substrates. *J. Phys.: Condens. Matter* **2013**, *25*, 095002. doi:10.1088/0953-8984/25/9/095002.
5. Xie, X.; Wang, H.; Wee, A.; Loh, K.P. The evolution of 3×3,6×6,$\sqrt{3}\times\sqrt{3}$R30° and $6\sqrt{3}\times6\sqrt{3}$R30° superstructures on 6H-SiC(0001) surfaces studied by reflection high energy electron diffraction. *Surf. Sci.* **2001**, *478*, 57–71. doi:10.1016/s0039-6028(00)01064-5.
6. Das, A.; Chakraborty, B.; Sood, A. Raman spectroscopy of graphene on different substrates and influence of defects. *Bull. Mater. Sci.* **2008**, *31*, 579–584.
7. Velez-Fort, E.; Mathieu, C.; Pallecchi, E.; Pigneur, M.; Silly, M.G.; Belkhou, R.; Marangolo, M.; Shukla, A.; Sirotti, F.; Ouerghi, A. Epitaxial graphene on 4H-SiC (0001) grown under nitrogen flux: Evidence of low nitrogen doping and high charge transfer. *ACS Nano* **2012**, *6*, 10893–10900.
8. Yang, R.; Huang, Q.; Chen, X.; Zhang, G.; Gao, H.J. Substrate doping effects on Raman spectrum of epitaxial graphene on SiC. *J. Appl. Phys.* **2010**, *107*, 034305.
9. Caffrey, N.M.; Armiento, R.; Yakimova, R.; Abrikosov, I.A. Charge neutrality in epitaxial graphene on 6 H-SiC (0001) via nitrogen intercalation. *Phys. Rev. B* **2015**, *92*, 081409.
